# Evolutionary and Phylogenetic Dynamics of SARS-CoV-2 Variants: A Genetic Comparative Study of Taiyuan and Wuhan Cities of China

**DOI:** 10.3390/v16060907

**Published:** 2024-06-03

**Authors:** Behzad Hussain, Changxin Wu

**Affiliations:** 1 Institutes of Biomedical Sciences, Shanxi University, 92 Wucheng Road, Taiyuan 030006, China; behzadhussain2@gmail.com; 2 Shanxi Provincial Key Laboratory of Medical Molecular Cell Biology, Shanxi University, 92 Wucheng Road, Taiyuan 030006, China; 3 The Key Laboratory of Chemical Biology and Molecular Engineering of National Ministry of Education, Shanxi University, 92 Wucheng Road, Taiyuan 030006, China; 4 Shanxi Provincial Key Laboratory for Major Infectious Disease Response, Taiyuan 030006, China; 5 The Fourth People’s Hospital of Taiyuan, Taiyuan 030006, China

**Keywords:** SARS-CoV-2, phylogenetic tree, phylogenetic network, evolutionary dynamics

## Abstract

Severe acute respiratory syndrome coronavirus 2 (SARS-CoV-2) is a positive-sense, single-stranded RNA genome-containing virus which has infected millions of people all over the world. The virus has been mutating rapidly enough, resulting in the emergence of new variants and sub-variants which have reportedly been spread from Wuhan city in China, the epicenter of the virus, to the rest of China and all over the world. The occurrence of mutations in the viral genome, especially in the viral spike protein region, has resulted in the evolution of multiple variants and sub-variants which gives the virus the benefit of host immune evasion and thus renders modern-day vaccines and therapeutics ineffective. Therefore, there is a continuous need to study the genetic characteristics and evolutionary dynamics of the SARS-CoV-2 variants. Hence, in this study, a total of 832 complete genomes of SARS-CoV-2 variants from the cities of Taiyuan and Wuhan in China was genetically characterized and their phylogenetic and evolutionary dynamics studied using phylogenetics, genetic similarity, and phylogenetic network analyses. This study shows that the four most prevalent lineages in Taiyuan and Wuhan are as follows: the Omicron lineages EG.5.1.1, followed by HK.3, FY.3, and XBB.1.16 (Pangolin classification), and clades 23F (EG.5.1), followed by 23H (HK.3), 22F (XBB), and 23D (XBB.1.9) (Nextclade classification), and lineage B followed by the Omicron FY.3, lineage A, and Omicron FL.2.3 (Pangolin classification), and the clades 19A, followed by 22F (XBB), 23F (EG.5.1), and 23H (HK.3) (Nextclade classification), respectively. Furthermore, our genetic similarity analysis show that the SARS-CoV-2 clade 19A-B.4 from Wuhan (name starting with 412981) has the least genetic similarity of about 95.5% in the spike region of the genome as compared to the query sequence of Omicron XBB.2.3.2 from Taiyuan (name starting with 18495234), followed by the Omicron FR.1.4 from Taiyuan (name starting with 18495199) with ~97.2% similarity and Omicron DY.3 (name starting with 17485740) with ~97.9% similarity. The rest of the variants showed ≥98% similarity with the query sequence of Omicron XBB.2.3.2 from Taiyuan (name starting with 18495234). In addition, our recombination analysis results show that the SARS-CoV-2 variants have three statistically significant recombinant events which could have possibly resulted in the emergence of Omicron XBB.1.16 (recombination event 3), FY.3 (recombination event 5), and FL.2.4 (recombination event 7), suggesting some very important information regarding viral evolution. Also, our phylogenetic tree and network analyses show that there are a total of 14 clusters and more than 10,000 mutations which may have probably resulted in the emergence of cluster-I, followed by 47 mutations resulting in the emergence of cluster-II and so on. The clustering of the viral variants of both cities reveals significant information regarding the phylodynamics of the virus among them. The results of our temporal phylogenetic analysis suggest that the variants of Taiyuan have likely emerged as independent variants separate from the variants of Wuhan. This study, to the best of our knowledge, is the first ever genetic comparative study between Taiyuan and Wuhan cities in China. This study will help us better understand the virus and cope with the emergence and spread of new variants at a local as well as an international level, and keep the public health authorities informed for them to make better decisions in designing new viral vaccines and therapeutics. It will also help the outbreak investigators to better examine any future outbreak.

## 1. Introduction

Severe acute respiratory syndrome coronavirus 2 (SARS-CoV-2) belongs to the subgenus sarbecovirus, under the genus *betacoronavirus*. It probably originated from Wuhan city in China and started infecting humans from December 2020 [1,2], giving rise to the coronavirus disease 2019 (COVID-19) which was declared a pandemic by the World Health Organization (WHO) in March 2020 [3]. It possibly originated from bats, as confirmed by the phylogenetic analysis studies showing a high similarity to the bat coronaviruses [4]. Globally, a total number of 773.8198 million people have been infected, with cumulative deaths of 7.0105 million people with this virus as of 31 December 2023 (https://covid19.who.int/; accessed on 20 March 2024). In China, from 3 January 2020 to 31 December 2023, a total of 99.3 million people have been infected, with cumulative deaths of 121,900, as reported to the WHO (https://covid19.who.int/region/wpro/country/cn; accessed on 20 March 2024).

SARS-CoV-2 is an enveloped, single-stranded, positive-sense RNA-containing virus with a genome size of approximately 29.9kb which encodes four structural proteins (SPs) (spike-S, envelope-E, membrane-E and nucleocapsid-N) and 16 non-structural proteins (NSPs) (NSP 1–16) [5,6]. In addition, it also encodes several accessory proteins, such as ORF3a-b, ORF6, ORF7a-b, ORF8, ORF9b-c and ORF10 [6].

While SARS-CoV-2 possesses a proofreading mechanism due to the exonuclease activity of NSP14 as studied by Gribble et al. 2021, the presence of a more conducive environment, and genetic recombination mechanisms, it has the advantage of rapidly evolving and resulting in the emergence of several new variants and subtypes like Alpha, Beta, Gamma, Delta, Kappa, Omicron, and others [7]. Within Omicron, there are many subtypes like Omicron BA.1, BA.2, BA.3, BA.4, BA.5, BQ.1.1, XBB.1.5, FY.3, EG.5.1, FU.1, HK.3, etc. [7,8,9,10,11]. The mutation rate of SARS-CoV-2 is ~1 × 10^−3^ nucleotide substitutions per year [12,13]. The presence of these mutations in the structural proteins, especially the spike protein, renders the anti-SARS-CoV-2 vaccines therapeutically ineffective [14]. All the information related to the full genome sequences of SARS-CoV-2 is available on the Global Initiative on Sharing All Influenza Data (GISAID) website, which helps the researchers in the relevant fields to study these emerging and rapidly evolving sequences to better understand the origin of the disease and the other aspects, including the phylogenetics and phylodynamics of the virus [15]. An in-depth genetic analysis of the complete genome of the SARS-CoV-2 variants is crucial for understanding the evolutionary dynamics of the virus. Many researchers have used different bioinformatics tools and techniques to study the genetic characteristics of the SARS-CoV-2 variants from different locations. These analyses are very important to ultimately control the viral spread [16,17,18,19]. While several studies have been conducted to investigate the genetic evolution and diversity of the SARS-CoV-2 variants on a global scale, there is a need to study the region-specific evolutionary dynamics of the virus. Hence, in this study, we characterized a total of 832 complete genomes of SARS-CoV-2 variants from two cities in China, Taiyuan and Wuhan, using lineage and subtyping analysis, phylogenetic tree, genetic similarity, and phylogenetic network analysis to help reveal a lot of useful information regarding the evolutionary dynamics of the viral variants, their genetic similarities/dissimilarities, and their phylogenetic relationships.

This comparative study, to the best of our knowledge, is the first study which genetically characterizes the complete genomes of SARS-CoV-2 variants from the cities of Taiyuan and Wuhan in China. With the help of this study, researchers can gain valuable insights into the evolutionary and underlying adaptation mechanisms of SARS-CoV-2 at the local level. This will help the public health facilities make better informed decisions for controlling the SARS-CoV-2 infection, and other viral infections in general, at an early stage of the epidemic by designing new vaccines based on the genetic characteristics of the viral variants. In addition, this study is particularly significant in countries like China where the virus has long been circulating, potentially leading to the emergence of new and unique variants and lineages with varying degrees of evolutionary trajectories.

## 2. Materials and Methods

### 2.1. Sequence Acquisition

On 7 December 2023, we retrieved FASTA sequence congregations along with their rational meta data from GISAID EpiCoV server (https://www.epicov.org/ accessed on 20 March 2024) [15] using the data filter as: virus name: hCoV-19, host: human, location: Asia/China/Shanxi/Taiyuan and Asia/China/Wuhan, complete, high coverage, clade: all. A total of 832 sequences were retrieved, of which 485 were from Wuhan and 347 from Taiyuan. The SARS-CoV-2 reference sequence (GenBank accession number: NC_045512.2) was accessed from NCBI database (https://www.ncbi.nlm.nih.gov/ accessed on 20 March 2024).

### 2.2. Genome Annotation

SARS-CoV-2 Wuhan-Hu-1 (Accession No.: NC_045512) genome annotation was performed using the Unipro UGENE v48.0 software (https://ugene.net/ accessed on 20 March 2024) [20] and the annotated genome was then presented circular form. 

#### Lineage and Subtyping Analysis

Clades and their Pango lineages of each of the sequence/isolate were determined using the Nextclade webserver v3.6.0 (https://clades.nextstrain.org/ accessed on 20 March 2024) and Pangolin (Phylogenetic Assignment of Named Global Outbreak Lineages) Webserver v4.3 (https://pangolin.cog-uk.io/ accessed on 20 March 2024). The Nextclade server used, by default, Wuhan-Hu-1/2019 (MN908947) as the reference sequence. After the lineages and subtypes were determined by the Pangolin and Nextstrain classification systems, the names of the sequences were modified as: Accession/EPI_ISL No.: Name of the virus/City-Year-Lineage name.

### 2.3. Sequence Alignment

All the sequences were aligned with a light-weight algorithm of Multiple Alignment using the Fast Fourier Transform v7 Web server called the FFT-NS-fragment method with rapid calculation of the full-length Multiple Sequence Alignment of closely related viral genomes (https://mafft.cbrc.jp/alignment/software/closelyrelatedviralgenomes.html accessed on 20 March 2024) [21]. The sequences were kept equal in size, with reference to the reference sequence. The multiple sequence alignment was then manually trimmed, where the ambiguous sequences and gaps were removed using BioEdit v7.2.5 (https://bioedit.software.informer.com/ accessed on 20 March 2024) [22] based on the best of our knowledge, while keeping the alignment as intact as possible for the most accurate analysis.

### 2.4. Phylogenetic Tree Construction

The Maximum Likelihood (ML) rooted phylogenetic tree was constructed using the IQ-Tree2 multicore v1.6.12 (http://www.iqtree.org/ accessed on 20 March 2024) [23] with the best-fit substitution model GTR+F+I+G4 (as determined by the ModelFinder tool built-in in IQ-Tree based on Bayesian Information Criterion—BIC), using 1000 bootstrap replicates and 1000 iterations [23]. The tree was rooted on the mid-point. The tree was then visualized and edited through the iTOL (Interactive Tree of Life) webserver v6 (https://itol.embl.de/ accessed on 20 March 2024) [24] by showing the bootstrap values at each node/branch of the tree and coloring all the branches of a subtype with a different color.

### 2.5. Time-Based Phylogenetic Tree Construction

The time-based phylogenetic tree was constructed based on the spike region of the SARS-CoV-2 multiple aligned genome sequences using the Bayesian Evolutionary Analysis Sampling Trees (BEAST) v1.10.4 software [25]. The multiple sequence alignment file was modified to write the time (dates in this case as: yyyy-mm-dd) at the end of each sequence name and the tip dates option was chosen in the Bayesian Evolutionary Analysis Utility (BEAUTi) program of the BEAST software. The GTR with gamma (=4) invariant sites substitution model was chosen, as previously determined by the IQ-Tree2 software and the strict clock with constant size coalescent tree model. Furthermore, the Markov chain Monte Carlo (MCMC) value of 10 million was used. The tree was then annotated with the TreeAnnotator v1.10.4 program of BEAST, and visualized and edited thereafter using the Interactive Tree of Life (iTOL) webserver v6 (https://itol.embl.de/ accessed on 20 March 2024). Median height was shown on each node and branch of the tree. The overall time-based trend of the variants was also checked by the Nextclade v3.6.0 webserver (https://clades.nextstrain.org/ accessed on 20 March 2024).

### 2.6. Genetic Similarity Analysis

The genetic similarity analysis was performed using SimPlot v3.5.1 (https://mybiosoftware.com/simplot-3-5-1-sequence-similarity-plotting.html accessed on 20 March 2024) [26]. The Kimura 2-parameter distance model was used with 1000 bootstrap replicates, Ts/Tv ratio of 2, and a neighbor-joining tree model. Window size and step size were set as 600 and 200, respectively, to obtain the best-looking results. One sequence from each of the clades was included in the analysis. The Omicron XBB.2.3.2 from Taiyuan (name starting with 18495234) was used as query sequence.

### 2.7. Recombination Analysis

Recombination Detection Program (RDP) version (http://web.cbio.uct.ac.za/~darren/rdp.html accessed on 20 March 2024) 5.29 [27] and Recombination Analysis Tool (RAT) (http://jic-bioinfo.bbsrc.ac.uk/bioinformatics-research/staff/graham_etherington/RAT.html accessed on 20 March 2024) [28] were used to detect recombination in the multiple sequence aligned full-length genomes of SARS-CoV-2. For RDP, seven algorithms, namely RDP, GENECONV, Bootscan, Chimaera, MaxChi, SiScan, and 3Seq, were used separately (because of the large burden on the bioinformatics software and the computer being used for the analysis) to identify any recombination event(s), if any.

### 2.8. Phylogenetic Network Analysis

Phylogenetic network analysis is a powerful way to infer the genetic relationships among the analyzed sequences. Hence, the multiple aligned sequences (*n* = 834, including the reference SARS-CoV-2 and bat coronavirus sequences) were evaluated by constructing the phylogenetic network using the Temporal Clustering of Sequences (TCS) method, which uses parsimony statistics to infer the network based on the number of mutations between sequences, implemented in the Population Analysis with Reticulate Trees (PopART) v1.7 software (http://popart.otago.ac.nz/ accessed on 20 March 2024) [29], where more than 5 percent of sites containing undefined subtypes were masked.

## 3. Results

### 3.1. Sequence Acquisition

We analyzed a total of 832 SARS-CoV-2 full-length genome sequences plus the wild-type reference genome and bat coronavirus genome (accessed from GISAID and NCBI database). The search filter location of Asia/China/Shanxi/Taiyuan resulted in a total number of 686 complete genomes of SARS-CoV-2, which was then narrowed down to 347 sequences by choosing the high coverage option. The search filter location of Asia/China/Hubei-Wuhan resulted in a total number of 485 complete sequences. The highest number of sequences were of 19-A-B (167), followed by EG.5.1.1 (157), HK.3 (126), FY.3 (97), and XBB.1.16.1.1 (27). Table 1 shows the details of the sequences used in this study.

### 3.2. Genome Annotation

**Figure 1:**
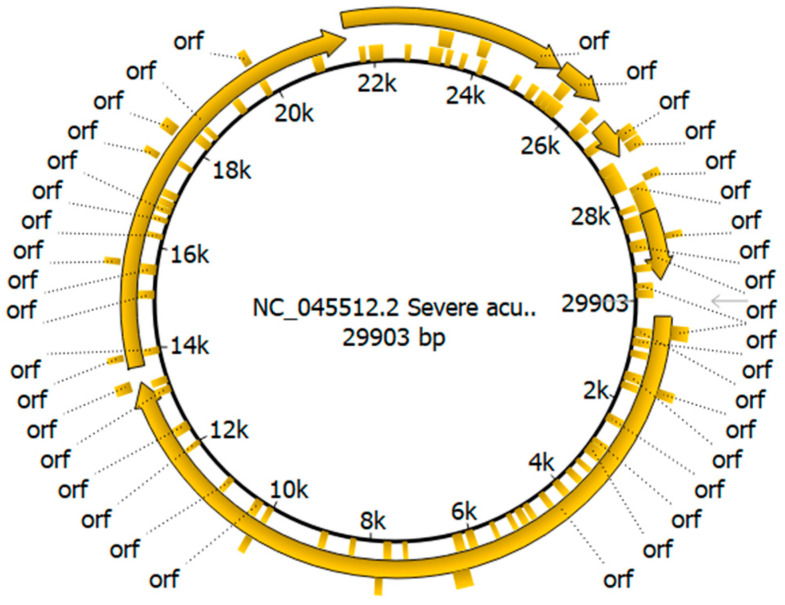
Genome annotation of SARS-CoV-2 Wuhan-Hu-1. The figure shows the circular form of the annotated genome of the SARS-CoV-2 original Wuhan strain (Wuhan-Hu-1; accession number: NC_045512). The complete genome is 29903 nucleotide bases in size. It shows different open reading frames (ORFs), including the spike region (from about 22 k to 24.5 k) which is one of the most important viral genes. The figure was created with the Unipro UGENE v48.0 software.

Genome annotation of SARS-CoV-2 Wuhan-Hu-1 (Accession No.: NC_045512) was carried out using the Unipro UGENE v48.0 software and the annotated genome was presented in a circular form in Figure 1. The annotated genome serves as a crucial reference for comparative genomic studies and gives us an understanding of the virus’ genomic features. It provides a framework to map genomic features like open reading frames (ORFs). It is essential for identifying genomic regions encoding various viral proteins (spike, nucleocapsid, etc.), comparing sequences of new SARS-CoV-2 variants to the reference to identify mutations and genomic changes over time, studying the evolution of the virus and designing diagnostic tests, vaccines, and therapeutic targets based on the reference sequence.

### 3.3. Subtype and Lineage Analysis

We determined and confirmed the subtype and lineage of the analyzed sequences through Nextclade and Pangolin classification, respectively. Due to the high burden of the sequences, the sequences of Taiyuan and Wuhan were analyzed separately, and their results were then combined (Table S1).

The four most prevalent lineages of SARS-CoV-2 in Wuhan as determined by Pangolin lineage classification are lineage B, followed by the Omicron FY.3, lineage A, and Omicron FL.2.3, whereas the four most prevalent clades in Wuhan as determined by Nextclade classification are the clades 19A, followed by 22F (XBB), 23F (EG.5.1), and 23H (HK.3).

The four most prevalent lineages in Taiyuan as determined by the Pangolin classification are the Omicron lineages EG.5.1.1, followed by HK.3, FY.3, and XBB.1.16, whereas the four most prevalent clades in Taiyuan as determined by Nextclade classification are the clades 23F (EG.5.1), followed by 23H (HK.3), 22F (XBB), and 23D (XBB.1.9).

### 3.4. Phylogenetic Tree

**Figure 2:**
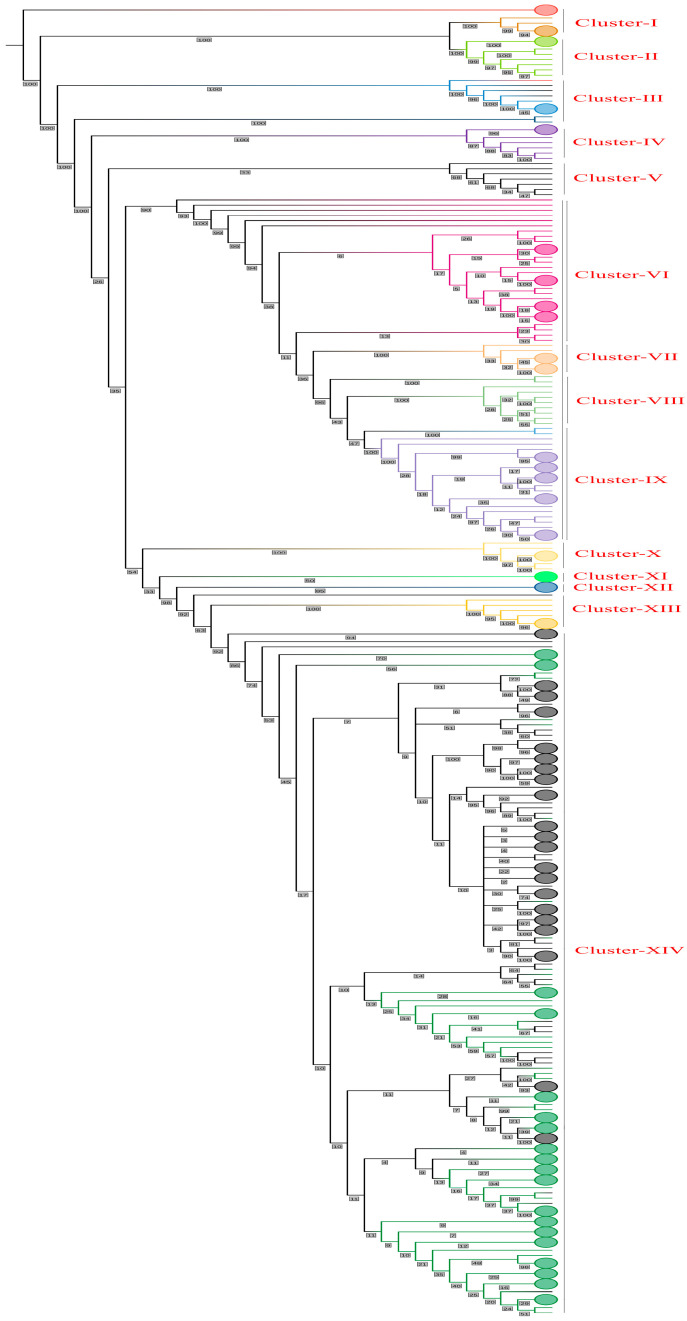
Maximum Likelihood (ML) Phylogenetic tree. The figure shows the maximum likelihood (ML) phylogenetic tree constructed using the IQ-Tree2 multicore software with the best-fit substitution model GTR+F+I+G4. The tree is rooted on the mid-point and shows 14 clusters containing the different variants of SARS-CoV-2. Each cluster is given a different color randomly. Some sequences have been collapsed to better visualize the tree. The tree was edited and visualized by the iTOL webserver v6. Bootstrap values have been shown on each node.

To check and determine the genetic relationship between Taiyuan and Wuhan, we performed a phylogenetic analysis using the complete genomes of SARS-CoV-2 from both cities. The phylogenetic tree showed some interesting relationships among the sequences. The tree shows that all the sequences of 2019 and 2020 (a total of 197 sequences from Wuhan as none of the sequences was from Taiyuan) including the subtypes/clades 19A-B, 19B-A, were grouped together as one big clade which was then collapsed. Some other less important sequences were collapsed together to make the tree visibly better. The tree was then divided into 14 clusters based on the topology and bootstrap values, as can be seen in (Figure 2 and Figure S1).

The Omicron BF.7.14, FR.1.1, GF.1, and XBB.1.16 from Taiyuan (names starting with 18495276, 18495161, 18495266 and 18495165) were shown to be more closely related to those from Wuhan (names starting with 17485739, 17672008, 17684335, and 17729945, respectively) but distantly related to those from Taiyuan itself (name starting with 18495416, 18495192, 18495282, and 18495390, respectively). The Omicron FU.2.1, FE.1.1, FY.3.1, FL.15, FL.2.4, EG.5.1, EG.5.1.4, HK.3, EG.5.1.1 from Taiyuan (names starting with 18495302, 18495297, 18495289, 18495442, 18495166, 18495417, 18495525, 18495522, 18495444) were closely related to those from Wuhan (names starting with 17978548, 18146040, 17837024, 17672010, 17801857, 18376489, 18284743, 18438428, 18254244, respectively) with bootstrap values of 100%. The Omicron EG.5.1 from Taiyuan (name starting with 18495387, 18495518, 18495440) was closely related to those from Wuhan (name starting with 17988088, 18146042, 18401765) with bootstrap values of 76%, 58%, and 38%, respectively. The Omicron EG.5.1.1 from Wuhan (name starting with 18254243) was more closely related to that of Taiyuan (name starting with 18495305) but distantly related to that of Wuhan itself (name starting with 18105544). The Omicron EG.5.1.1 from Taiyuan (names starting with 18495407, 18495433, 18495438, 18495170, 18495338, and 18495286) were more closely related to those from Wuhan (names starting with 18146047, 18254254, 18146060, 18078299, 17978550, and 18254247) with 50%, 23%, 22%, 16%, and 15% bootstraps, respectively. The Omicron FL.2.3 from Taiyuan (names starting with 18495201, 18495361, 18495184) were more closely related to that from Wuhan (names starting with 17729943, 17672006, 17684329, respectively) but with relatively lower bootstrap values of 34%, 50%, 62%, respectively. The Omicron FL.4 from Taiyuan (name starting with 18495316) was closely related to that of the Wuhan (name starting with 17794191) with a bootstrap value of 98%. The Omicron XBB.1.9.1 from Taiyuan (name starting with 18495200) was closely related to that of Wuhan (name starting with 17672025) but with a bootstrap value of only 25%. The Omicron XBB.1.9.1 from Taiyuan (name starting with 18495306) was closely related to the Omicron FL.18 from Wuhan (name starting with 17684330) but only with an 18% bootstrap. The Omicron FY.3 from Taiyuan (name starting with 18495283) was closely related to the Omicron XBB.1.9.2 from Taiyuan (name starting with 18495281) with a bootstrap of 94%. The Omicron HK.3 from Wuhan and Taiyuan (names starting with 18146054 and 18495477, respectively) were closely related with the Omicron EG.5.1.1 from Wuhan and Taiyuan (names starting with 18105541 and 18495317 respectively) with 100% and 55% bootstraps, respectively. The Omicron HK.3 from Taiyuan (names starting with 18495409, 18495504) were closely related with those from Wuhan (names starting with 18146053, 18535381, respectively) with bootstraps of 30% and 67%, respectively. The Omicron HK.2 from Taiyuan (name starting with 18495464) was closely related with those from Wuhan (name starting with 17978544) with a bootstrap of 49%. The Omicron HK.1 from Taiyuan (name starting with 18495398) was closely related with the Omicron EG.5.1.1 from Wuhan (name starting with 18105543) with 99% bootstraps. One of the Omicron XBB.1.5 sequences from Wuhan (name starting with 17978557) showed more genetic similarity with the Omicron GR.1 from Wuhan (name starting with 17729936) than with the other Omicron XBB.1.5 from Wuhan (name starting with 17729949). The Omicron DY.2 from Taiyuan (name starting with 18495304) was shown to be more closely related to Omicron BA.5.2.48 from Taiyuan (name starting with 18495357) but distantly related to those from Taiyuan itself (names starting with 18495303 and 18495280).

### 3.5. Time-Based Phylogenetic Tree

**Figure 3:**
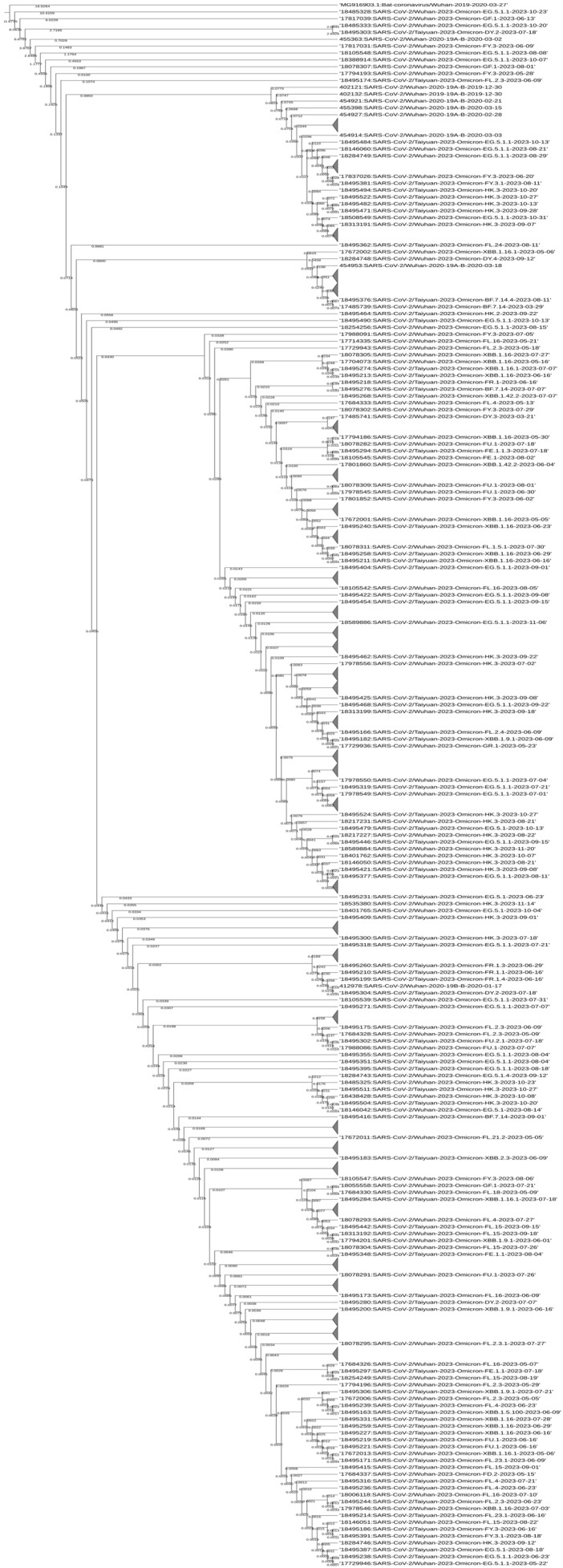
Time-based phylogenetic tree. The figure shows the time-based phylogenetic tree constructed using BEAST software with the substitution model GTR+F+I+G4. Some sequences have been collapsed to better visualize the tree. The tree was edited and visualized by iTOL webserver v6. The median height values are shown on each node and branch of the tree to indicate the time of distribution of each variant among the two cities.

The time-based phylogenetic tree showed a temporal distribution of the SARS-CoV-2 variants. Interestingly, the comparative analysis of Taiyuan and Wuhan showed that some variants of Taiyuan are more closely related to those of Wuhan rather than to Taiyuan itself, and they would have originated later in time as can be seen from the median height; for example, Omicron FE.1.1.3 and FU.2.1 from Taiyuan (name starting with 18495294 and 18495302, respectively) were shown to be more closely related to the Omicron FE.1 and FU.1 from Wuhan (name starting with 18105545 and 17988086, respectively), respectively, and were shown to have originated later in time. A detailed comparison of the sequences from the two cities can be seen in Figure 3 and Figure S2.

**Figure 4:**
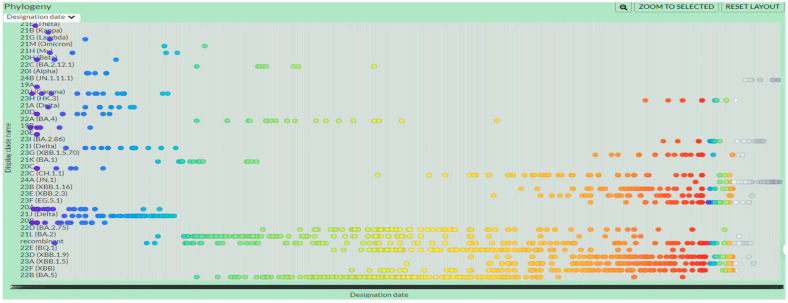
Overall temporal trend of the variants. The figure shows the overall trend of the SARS-CoV-2 variants based on time. The graph shows the designation date on the X-axis and clade name on the Y-axis. From left to right, the X-axis shows the number of years from 2020 to 2024, and each colored dot represents an individual genome sequence with a particular year. The dark blue and purple dots show the genome sequences of year 2020–2021 followed by the light blue and aqua dots (year 2021), light green dots (year 2022), yellow and orange dots (year 2023), red dots (later time in the year 2023) and the gray dots (year 2024). As the analysis was conducted based on the spike region, some sequences might have been misidentified because of the inherent errors of the classification system. The colors have been assigned randomly by the webserver.

The overall time-based trend of the SARS-CoV-2 variants of the two cities was analyzed and checked by the Nextclade webserver v3.6.0, and the temporal trend can be seen in Figure 4.

### 3.6. Genetic Similarity Analysis

**Figure 5:**
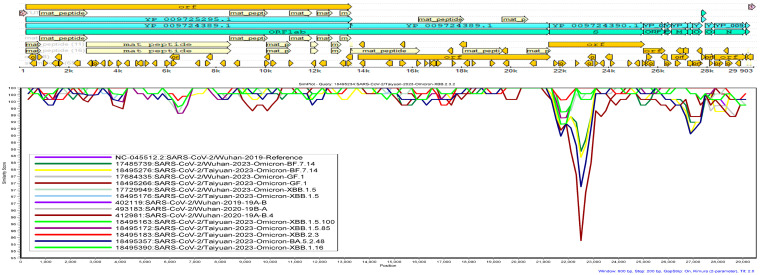
Genetic similarity plot of representative SARS-CoV-2 variants. The figure shows the genetic similarity plots of the selected variants of SARS-CoV-2 in comparison with the Omicron XBB.2.3.2 from Taiyuan (18495234) as query sequence. Genetic similarity (%) has been shown on the Y-axis and the nucleotide position has been shown on the X-axis. Clade 19A-B.4 from Wuhan (412981) has the lowest genetic similarity of about 95.5% between the nucleotide position 22000 and 24000, which is the spike protein’s nucleotide position. Most of the variants show a genetic similarity of 98% or more.

The genetic similarity plot shows that the SARS-CoV-2 clade 19A-B.4 from Wuhan (name starting with 412981) has the lowest genetic similarity (that is about 95.5%) between the nucleotide position 22000 and 24000; that is the spike protein nucleotide position compared to the query sequence of Omicron XBB.2.3.2 from Taiyuan (name starting with 18495234), followed by the Omicron FR.1.4 from Taiyuan (name starting with 18495199) (~97.2% similarity), and Omicron DY.3 (name starting with 17485740) with ~97.9% similarity. The rest of the variants show equal to or more than 98% similarity with the query sequence of Omicron XBB.2.3.2 from Taiyuan (name starting with 18495234), as can be seen in Figure 5.

### 3.7. Recombination Analysis

Genetic recombination plays a crucial role in virus evolution resulting in the emergence of new variants of SARS-CoV-2. It helps us to better understand the evolution and genetic diversity of the virus. In our study, we analyzed 832 complete genomes of SARS-CoV-2 from Wuhan and Taiyuan for any potential recombination event (s). A total of nine recombination events were detected by the RDP5 software v5.29 as can be seen in Table 2 and Table S2.

Out of nine recombination events detected, only three (Event Nos. 3, 5, and 7) were shown to be verified by four or more detection methods implemented in the RDP5 software but all these events were at the 3′ end of the genome.

Event 3 was verified by five detection methods and showed that the minor parent was 19B-A of Wuhan (455406) while the major parent was unknown (but the software showed the most probable major parent, i.e., Omicron-FL.13.1 of Wuhan (17672007)), resulting in the recombinant Omicron-XBB.1.16 of Wuhan (17672023). Event 5 was verified by six detection methods and showed that the minor parent was 19A-B of Wuhan (454997), and the major parent was unknown (but the software showed the most probable major parent, i.e., Omicron-FY.3 of Wuhan (17978543)), resulting in the recombinant Omicron-FY.3 of Wuhan (17672021). Event 7 was also verified by five detection methods and showed that the minor parent was Omicron-DY.4 from Wuhan (18284748), and the major parent was unknown (but the software showed the most probable major parent, i.e., Omicron-FD.2 of Wuhan (17684337)), resulting in the recombinant Omicron-FL.2.4 of Wuhan (17801857).

Interestingly, Event 8 showed that the minor parent was Omicron FR.1-Taiyuan (18495199) and the major parent was Omicron EG.5.1.1-Taiyuan (18495364), resulting in the recombinant Omicron EG.5.1-Taiyuan (18495231). Also, only Event 8 showed recombination at the spike protein nucleotide positions, i.e., around 22,000 nucleotide positions in the alignment but this event was verified by only one detection method.

### 3.8. Phylogenetic Network Analysis

Due to the presence of reticulate evolutionary phenomena, for example, recombination, the evolution of many species cannot be inferred using phylogenetic trees. Hence, phylogenetic network analysis was performed to better understand the evolution of the SARS-CoV-2 variants between the Wuhan and Taiyuan cities in China.

A total of 832 sequences, plus the reference and bat coronavirus sequences, multiple aligned complete genomes of SARS-CoV-2 were analyzed by constructing a phylogenetic network using the Temporal Clustering of Sequences (TCS) method. The analysis showed that there is a total of 11,466 segregating sites, including 1000 parsimony-informative (PI) sites. The sequences of the SARS-CoV-2 variants were then distributed based on the number of mutations. The details of the network are given in Table 3. The negative value (−2.33398) of Tajima’s D statistic shows that the segregating and PI sites are statistically significant in the evolution of the viral target genomes.

The phylogenetic network of the SARS-CoV-2 genomes probably originated from the bat-CoV, with a result of more than 10,000 mutations. The network was then divided into 14 clusters as follows: Cluster I-XIV, which was consistent with the phylogenetic tree. Cluster-I contains all the sequences of 2019–2020 from Wuhan, i.e., subtypes A, B, and B.4, including the wild-type (reference) sequence. Cluster-II has probably been originated from Cluster-I because of a total of 47 mutations, and it also contained the sequences of Omicron DY.2/3, BF, and, interestingly, the Omicron BA.5.2.48. Cluster-III contained Omicron FR with one Omicron BN sequence.

**Figure 6:**
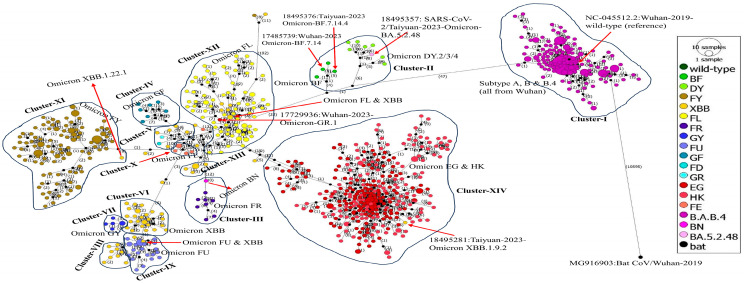
Phylogenetic network of SARS-CoV-2 variants from Taiyuan and Wuhan. The figure shows the phylogenetic network of all the 832 complete genomes of SARS-CoV-2 from Taiyuan and Wuhan, plus the reference and bat-CoV genomes, constructed by the PopArt v1.7 software. The network was constructed using the TCS method and shows that the SARS-CoV-2 genomes probably originated from the bat-CoV with a result of more than 10,000 mutations. The network was divided into 14 clusters, consistent with the phylogenetic tree. The numbers indicate the number of mutations, and each color represents a different main variant. Some interesting variants have been labeled on the network.

Cluster-IV contained the sequences of Omicron GF, while cluster-V contained a few sequences of Omicron XBB and one of each of the Omicron FD and GR. Cluster-VI and VIII contained the sequences of Omicron XBB, while Cluster-VII contained the sequences of Omicron GY. Cluster-VIX contained the sequences of Omicron FU, while Cluster-X contained the sequences of Omicron FE. Cluster-XI contained the sequences of Omicron FY with one Omicron XBB.1.22.1 from Taiyuan (name starting with 18495245), while Cluster-XII contained the sequences of Omicron FL from both Taiyuan and Wuhan, with one Omicron GR.1 from Wuhan (name starting with 17729936). Cluster-XIII contained some of the sequences of Omicron XBB, while Cluster-XIV contained the sequences of Omicron EG and HK, with a sequence of Omicron XBB.1.9.2 from Taiyuan (name starting with 18495281). The phylogenetic network analysis results are consistent with the phylogenetic tree. The number of mutations between each genome/variant and cluster of the phylogenetic network can be seen in Figure 6. 

## 4. Discussion

During the last two to three years, a lot of research has been conducted on the evolution of SARS-CoV-2 using different bioinformatics tools and techniques. Due to the mutations in SARS-CoV-2, multiple variants and lineages have originated. Regular monitoring of these mutations and tracking of the travel history between and within a particular city or location is very important to unveil the true route of viral evolution and for a better understanding of the comparative genetic characteristics between two locations or cities.

### 4.1. Subtype and Lineage Analysis

The subtyping and lineage analysis of our study shows that the most predominant lineage in both Wuhan and Taiyuan cities of China is Omicron and its sub-lineages. The emergence and prevalence of FY.3, FL.2.3, EG.5.1.1, HK.3, and XBB.1.16 Omicron sub-lineages is consistent with the global trend of SARS-CoV-2 (https://www.who.int/activities/tracking-SARS-CoV-2-variants accessed on 20 March 2024) [30,31,32].

### 4.2. Phylogenetic Analysis

The phylogenetic analysis revealed that all the 197 sequences from 2019 and 2020, exclusively from Wuhan, China, were grouped together in one clade. This grouping included subtypes/clades 19A-B and 19B-A, both of which formed a significant clade that was collapsed to better visualize the tree. Some of the less important sequences were also collapsed to enhance the tree’s visibility. These results align with a study on the transmission dynamics of SARS-CoV-2 in Wuhan, in which Sun and colleagues highlighted the significance of lockdowns and medical resources in controlling the spread of the virus using mathematical modelling [33]. In addition, another group of researchers studied the spatial distribution characteristics of SARS-CoV-2 in China using spatial analysis methods [34].

The close relatedness of the SARS-CoV-2 variants from Taiyuan and Wuhan indicates a shared evolutionary history. It is crucial for tracking the transmission dynamics of the SARS-CoV-2 variants between Taiyuan and Wuhan. Our results provide valuable data for researchers and healthcare departments to carefully monitor the evolution of SARS-CoV-2, which can help in controlling the spread of SARS-CoV-2 in particular and other viruses in general [35,36].

Interestingly, some sequences of SARS-CoV-2 from Taiyuan showed a closer genetic similarity with those from Wuhan, the epicenter of the initial outbreak, rather than those of the other SARS-CoV-2 sequences from Taiyuan. For example, the Omicron DY.2 from Taiyuan (name starting with 18495304) was shown to be more closely related to Omicron BA.5.2.48 but distantly related to those from Taiyuan itself. The reason might be that the Omicron BA.5.2.48 sequence has not been correctly classified by the Pangolin and Nextclade classifications. Also, the Omicron HK.1 from Taiyuan (name starting with 18495398) was shown to be closely related with the Omicron EG.5.1.1 from Wuhan (name starting with 18105543) with 99% bootstraps. The high bootstrap value suggests that both sequences might share a common epidemiological link or transmission route. It is probable that the individuals infected from these viral variants from Taiyuan and Wuhan were exposed to the same or similar infection source. In addition, it also suggests that the Omicron HK.1 from Taiyuan and Omicron EG5.1.1 from Wuhan have relatively low genetic divergence, which might indicate a relatively recent introduction with limited genetic mutations.

### 4.3. Time-Based Phylogenetic Analysis

Some variants of SARS-CoV-2 identified from Taiyuan were found to be more closely related to those from Wuhan rather than those within Taiyuan itself. This suggests that these variants from Taiyuan may have originated from or been introduced by the variants of Wuhan, rather than evolving independently within Taiyuan. The median heights of the time-based phylogenetic tree branches and nodes represent the probable origin and distribution of the SARS-CoV-2 variants, suggesting that the variants of Taiyuan emerged after the Wuhan variants that they are more closely related to. The results of our study highlight the importance of time-based phylogenetic analysis in giving us a better understanding of the evolutionary dynamics of the viral variants across different geographical locations. This can help with tracking the future spread of the viral variants across different regions for a better understanding of any future pandemic. There are many researchers who studied the temporal trends of the SARS-CoV-2 variants [37,38]. 

### 4.4. Genetic Similarity Analysis

The genome of SARS-CoV-2 has a positive-sense RNA of ~29.9 kb in size (Wu et al., 2020). The viral genomic RNA encodes nonstructural proteins (nsps) from two open reading frames (ORFs) called ORF1a and ORF1b. A large polyprotein is then produced from ORF1b, which is then cleaved to produce 15nsps. Shorter subgenomic RNAs (sgRNAs) are also produced which encode the spike (S), envelope (E), membrane (M), and nucleocapside (N) structural proteins. The genome is flanked by the 5′ and 3′ untranslated regions (UTRs) [1].

The clade 19A-B.4 from Wuhan (name starting with 412981) has the lowest genetic similarity compared to the Omicron XBB.2.3.2 from Taiyuan (name starting with 18495234) at nucleotide positions 22,000–24,000, the region which encodes the spike protein. This means that the former is more genetically distinct from the latter. This suggests significant evolutionary divergence between the two. This difference might contribute to different phenotypic characteristics that warrant further research. The variants showing 98% or more genetic similarity might exhibit more similar genetic and biological characteristics.

### 4.5. Recombination Analysis

A lot of researchers have developed different methods to check for recombination in SARS-CoV-2. Most of the time, recombination occurs in the spike gene, near its 5′ end [39,40]. The major and minor parents of a recombinant SARS-CoV-2 can be from both the 5′-end as well as 3′-end of the viral genome [41]. Recombination can happen between different variants or subtypes. In our study, we analyzed the complete genomes of SARS-CoV-2 from Wuhan and Taiyuan, China, for any potential recombination.

Interestingly, in Event 8, the minor parent was Omicron FR.1-Taiyuan (18495199), and major parent was Omicron EG.5.1.1-Taiyuan (18495364), resulting in the recombinant Omicron EG.5.1-Taiyuan (18495231); however, only one detection method detected this recombination event. Reasons for this might include the wrong assignment of the lineage names, and the convergent evolution by which the Omicron EG.5.1 may have independently acquired similar genetic changes to those found in the Omicron EG.5.1.1 rather than the parent–offspring relationship [42]. In addition, recombination Event 8 was shown to occur at the spike protein, at around 22,000 nucleotide positions, which is particularly significant. The spike protein is the key target for therapeutics and vaccine design, and a mutation/recombination in this region can affect the virus–host interaction, giving the virus the benefit of immune escape as well as affecting the vaccine efficacy.

### 4.6. Phylogenetic Network Analysis

The evolution of genes is tree-like, but the evolution of species is no longer tree-like because of the phenomena of recombination, horizontal gene transfer, and hybridization. In our phylogenetic network analysis, we used the Temporal Clustering of Sequences (TCS) method, which showed a negative value of Tajima’s D statistic that indicated changes in the viral population, possibly influenced by genetic drift, natural selection, and/or other mutations. This is consistent with the study of Al-Jawabreh and colleagues, but they constructed a phylogenetic network analysis using the median-joining method instead [43]. In addition, the occurrence of a significant PI and segregating sites shows genetic and evolutionary dynamics within the genomes of SARS-CoV-2 between the two cities, indicating the ongoing genetic diversity and adaptation of the virus. 

Our phylogenetic network analysis showed fourteen (XIV) clusters of SARS-CoV-2 genomes based on their mutations which align with our phylogenetic tree. Al-Jawabreh and colleagues constructed a median-joining phylogeographic network and reported three clusters of SARS-CoV-2 based on different geographical locations [40]. These analyses can help us better understand the relatedness between different SARS-CoV-2 variants from Wuhan and Taiyuan, which can help the public health authorities to effectively control the spread of the virus and mitigate its emerging mutants using targeted vaccine design and other surveillance strategies (https://cov-lineages.org/, accessed on 20 March 2024) [44,45].

### 4.7. Importance of the Study

The current study provides a comprehensive genetic characterization and analysis of SARS-CoV-2 variants from the cities of Taiyuan and Wuhan in China. This study is the first genetic comparative study between Taiyuan and Wuhan, to the best of our knowledge. The study identifies the most prevalent lineages and clades of the SARS-CoV-2 variants circulating between the two cities. It also provides valuable insights into the evolutionary dynamics and mechanisms driving the diversification and distribution of the SARS-CoV-2 variants. In addition, this study sheds light on the probable origin of SARS-CoV-2 variants of Taiyuan independently from those of the Wuhan variants. This study contributes to a better understanding of the SARS-CoV-2, its spread, and evolution at a local level, which can be extrapolated to an international level and will then help the public healthcare authorities in controlling the present and future epidemics and outbreaks by allowing them to design more targeted and effective therapeutics, vaccines, and outbreak investigation strategies.

## 5. Conclusions

From our study, it is concluded that there is a continuous need to study the genetic characteristics of the SARS-CoV-2 variants and other viruses as the rapidly mutating viral variants can render modern-day vaccines and therapeutics ineffective. It has been reported in this comparative study that SARS-CoV-2 has evolved over time and spread from the epicenter, Wuhan city, in China, to Taiyuan city. Many significant things were determined including, for example, the presence of multiple recombination events and clustering of viral variants from both cities as some viral variants from Taiyuan shared more similarity with those from Wuhan rather than with those from Taiyuan itself, and the recombination of Omicron EG.5.1 from Taiyuan as a result of potential convergent evolution. This study will help the healthcare and scientific community better understand the genetic characteristics, phylodynamics, and evolutionary pathways of the SARS-CoV-2 variants between local populations of China and the whole world.

## Figures and Tables

**Table 1:** Sequences used in this study.

SARS-CoV-2 Variant	City Name *	Total No. of Sequences
BF.7.14	T (2), W (1)	3
DY.3	W (2)	2
FY.3	T (28), W (69)	97
XBB.1.16.1	T (16), W (11)	27
XBB.1.16.1.1	T (7), W (3)	10
FL.2.3	T (7), W (14)	21
FL.13.1	W (1)	1
FR.1.1	T (4), W (1)	5
GY.1	T (3), W (3)	6
FL.15	T (2), W (7)	9
FL.21.2	W (1)	1
FU.1	T (9), W (13)	22
XBB.1.9.1	T (3), W (2)	5
XBB.1.16.18	W (2)	2
FL.16	T (3), W (4)	7
FL.18	W (1)	1
FL.4	T (9), W (4)	13
GF.1	T (2), W (5)	7
FD.2	W (1)	1
FL.2.4	T (4), W (2)	6
GR.1	W (1)	1
EG.5.1.1	T (113), W (44)	157
XBB.1.5	T (1), W (2)	3
HK.5	W (1)	1
FL.2	T (3), W (3)	6
FL.21	W (1)	1
EG.5.1	T (9), W (5)	14
FY.3.1	T (12), W (9)	21
XBB.1.42.2	T (4), W (1)	5
DY.4	W (2)	2
HK.1	T (1), W (1)	2
HK.2	T (4), W (1)	5
FU.2.1	T (1), W (1)	2
XBB.1.42	W (1)	1
HK.3	T (68), W (58)	126
FL.2.3.1	W (2)	2
FL.13.2	W (1)	1
FL.1.5.1	W (1)	1
FE.1	W (1)	1
FE.1.1	T (3), W (1)	4
HK.4	W (4)	4
EG.5.1.4	T (2), W (1)	3
19A-B	W (167)	167
19B-B	W (6)	6
19B-A	W (20)	20
19A-B.4	W (3)	3
XBB.1.5.100	T (1)	1
FL.23.1	T (3)	3
XBB.1.5.85	T (1)	1
XBB.2.3	T (1)	1
FR.1.4	T (1)	1
XBB.1.17.1	T (1)	1
FR.1	T (1)	1
BN.1.3.5	T (1)	1
FL.4.6	T (1)	1
XBB.1.9.2	T (2)	2
FL.5	T (2)	2
XBB.2.3.2	T (1)	1
XBB.1.22.1	T (1)	1
FR.1.3	T (1)	1
FL.24	T (3)	3
DY.2	T (3)	3
FE.1.1.3	T (1)	1
BA.5.2.48	T (1)	1
BF.7.14.4	T (1)	1

* T = Taiyuan city; W = Wuhan city.

**Table 2:** Identification of potential recombination events in the complete genome of SARS-CoV-2 variants.

Event	Recombinant	Minor Parent	Major Parent	Detection * (RGBMCST)
1	17672021:SARS-CoV-2/Wuhan-2023-Omicron-FY.3	Unknown (455406:SARS-CoV-2/Wuhan-2020-19B-A)	17684330:SARS-CoV-2/Wuhan-2023-Omicron-FL.18	−−−−−−−
2	17684330:SARS-CoV-2/Wuhan-2023-Omicron-FL.18	17729935:SARS-CoV-2/Wuhan-2023-Omicron-XBB.1.16	17672023:SARS-CoV-2/Wuhan-2023-Omicron-XBB.1.16	−−−−−−−
3	17672023:SARS-CoV-2/Wuhan-2023-Omicron-XBB.1.16	455406:SARS-CoV-2/Wuhan-2020-19B-A	Unknown (17672007:SARS-CoV-2/Wuhan-2023-Omicron-FL.13.1)	−+−++++
4	17684330:SARS-CoV-2/Wuhan-2023-Omicron-FL.18	Unknown (455406:SARS-CoV-2/Wuhan-2020-19B-A)	17672007:SARS-CoV-2/Wuhan-2023-Omicron-FL.13.1	-−−−−−−+
5	17672021:SARS-CoV-2/Wuhan-2023-Omicron-FY.3	454997:SARS-CoV-2/Wuhan-2020-19A-B	Unknown (17978543:SARS-CoV-2/Wuhan-2023-Omicron-FY.3)	−++++++
6	17801857:SARS-CoV-2/Wuhan-2023-Omicron-FL.2.4	Unknown (17672023:SARS-CoV-2/Wuhan-2023-Omicron-XBB.1.16)	17672007:SARS-CoV-2/Wuhan-2023-Omicron-FL.13.1	−+−−−−−
7	17801857:SARS-CoV-2/Wuhan-2023-Omicron-FL.2.4	18284748:SARS-CoV-2/Wuhan-2023-Omicron-DY.4	Unknown (17684337:SARS-CoV-2/Wuhan-2023-Omicron-FD.2)	−+−++++
8	18495231:SARS-CoV-2/Taiyuan-2023-Omicron-EG.5.1	18495199:SARS-CoV-2/Taiyuan-2023-Omicron-FR.1.4	18495364:SARS-CoV-2/Taiyuan-2023-Omicron-EG.5.1.1	−−−−−−+
9	17801857:SARS-CoV-2/Wuhan-2023-Omicron-FL.2.4	455406:SARS-CoV-2/Wuhan-2020-19B-A	Unknown (17729949:SARS-CoV-2/Wuhan-2023-Omicron-XBB.1.5)	−−−−−−+

* Detection methods: R, RDP; G, GENECONV; B, BootScan; M, MaxChi; C, Chimaera; S, SiScan; T, 3Seq. ‘+’ = verified; ‘−’ = not verified.

**Table 3:** Phylogenetic network analysis results.

Sr. No.	Network Method	Nucleotide Diversity	Segregating Sites	PI Sites *	Tajima’s D Statistic
1	TCS network	pi = 0.0142049	11466	1000	D = −2.33398; p (D ≥ −2.33398) = 0.999236

* PI sites = parsimony informative sites.

## Data Availability

The original contributions presented in the study are included in the article/Supplementary Material, further inquiries can be directed to the corresponding and the first author.

## Supplementary Materials

The following supporting information can be downloaded at: https://www.mdpi.com/article/10.3390/v16060907/s1, Figure S1: Mid-point rooted Tree; Figure S2: Time-based detailed phylogenetic tree; Table S1: Pangolin classification; Table S2: Recombination.
